# Does growth hormone supplementation improve oocyte competence and IVF outcomes in patients with poor embryonic development? A randomized controlled trial

**DOI:** 10.1186/s12884-020-03004-9

**Published:** 2020-05-20

**Authors:** Jingyu Li, Qiaoli Chen, Jiang Wang, Guoning Huang, Hong Ye

**Affiliations:** Chongqing Key Laboratory of Human Embryo Engineering, Chongqing Reproduction and Genetics Institute, Chongqing Health Center for Women and Children, No.64 Jin Tang Street, Yu Zhong District, Chongqing, 400013 China

**Keywords:** Growth hormone, In vitro fertilization, Poor embryonic development, Oocyte competence, Mitochondrial DNA

## Abstract

**Background:**

Many studies have demonstrated the benefits of the addition of growth hormone (GH) to the controlled ovarian stimulation protocol in vitro fertilization (IVF) cycles in poor-respond patients, but the effect of GH on patients with poor embryonic development remain unclear. This paper was designed to investigate the efficacy of GH co-treatment during IVF for the patients with poor embryonic development.

**Method:**

A randomized controlled trial including 158 patients with poor embryo development was conducted between July 2017 and February 2019. One hundred and seven patients were randomized for GH treatment (GH group) and 51 patients for untreated (control group). The primary end-points were the clinical pregnancy and live birth rates in the two groups. The oocyte competence were assessed through calculating the mitochondrial DNA (mtDNA) copy number in corresponding cumulus granulosa cells (CGCs). Quantitative PCR were used for calculation of mtDNA copy number.

**Results:**

Relative to the control group, GH co-treatment resulted in a significantly higher number of retrieved oocytes (10.29 ± 5.92 versus 8.16 ± 4.17, *P* = 0.023) and cleaved embryos (6.73 ± 4.25 versus 5.29 ± 3.23, *P* = 0.036). The implantation rate, clinical pregnancy rates per cycle, and live birth rate per cycle were higher in the GH group than in the control group (36.00% versus 17.86%, *P* = 0.005; 43.93% versus 19.61%, *P* = 0.005; 41.12% versus 17.65%, *P* = 0.006). CGCs of the GH group had significantly higher mtDNA copy numbers than CGCs of the control group (252 versus 204, *P* < 0.001).

**Conclusions:**

These data provided further evidence to indicate that GH supplementation may support more live births during IVF, in patients with poor embryonic development. It also appears that oocytes generated under GH co-treatment have a better developmental competence.

**Trial registration:**

ChiCTR1900021992 posted March 19, 2019 (retrospectively registered).

## Background

Poor embryonic development is a major cause of in vitro fertilization (IVF) failure, especially in patients with normal ovarian response. A strong association is known to exist between embryo quality and pregnancy rates [1, 2]. In the period following fertilization, no transcription occurs in the newly formed embryo [3, 4]; thus, early embryonic development is controlled by maternally derived information until the oocyte-to-embryo transition (OET) occurs. Indeed, maternal stores of mRNAs, proteins, and mitochondria are essential for fertilization, early embryonic development, and implantation [5, 6]. Therefore, the quality of the oocytes is a key factor in determining the quality of day-3 embryos that are ready for transfer. Several factors have been investigated in relation to reductions in oocyte developmental competence, including advanced age [7], an immature cytoplasm [8], abnormal maternal protein expression [9], and insufficient mitochondria [10]. However, to date, no treatment has successfully improved the clinical outcomes in patients with poor embryonic development.

A successful outcome of IVF is largely dependent on the number and quality of oocytes retrieved during the treatment cycle. Defining oocyte quality is not straightforward, but typically, the quality and developmental potential of oocytes are evaluated via simple morphological assessment [11, 12]. However, this evaluation cannot determine the status of predictive metabolic and mitochondrial molecular parameters [11]. Therefore, many oocytes that are defined as high quality by morphological assessment are not actually very developmentally competent, especially for implantation. In addition to morphological assessment, biochemical evaluations are necessary to accurately assess the true developmental competence of the oocyte [13–16].

Mitochondria have important roles in oocyte maturation [17], fertilization, and early embryonic development [18, 19]. Sufficient numbers of mitochondria are necessary to support the consumption of adenosine triphosphate (ATP) that occurs during the processes of early embryonic development [20, 21]. In humans, oocytes of women with ovarian insufficiency reportedly have lower mtDNA copy numbers than those of women with a normal ovarian profile [22], and unfertilized oocytes have lower mtDNA copy numbers compared to fertilized oocytes [18]. However, directly assessing the mtDNA copy number is not feasible, because it results in destruction of the oocyte. Examination of the cumulus granulosa cells (CGCs) that surround the oocyte is an effective alternative to the direct evaluation of oocytes [13, 14, 16, 23, 24], because the mtDNA copy number in CGCs correlates with embryo quality and implantation in IVF procedures [23, 24]. Consequently, assessment of the mtDNA content of CGCs provide a promising means of assessing oocyte developmental competence.

Growth hormone (GH; an anabolic peptide hormone) is an important regulator of ovarian steroidogenesis [25], follicular development [26] and oocyte maturation [27]. The physiological effect of GH on the oocyte and folliculogenesis is presumed to be via insulin-like growth factor 1 (IGF-1) or by a direct action of GH. GH can stimulate serum and follicular IGF-1 [28]. Thus, the rationale of GH supplemented into controlled ovarian stimulation protocol is that the follicular fluid IGF-1 concentrations of women undergoing IVF is directly correlated to the number of developing follicles [29]. In addition, follicular concentrations of GH have been shown displayed a positive correlation to oocyte competence, including fertilization, embryonic development, embryo implantation, and clinical pregnancy [30, 31]. In animal models, GH can improve oocyte cytoplasmic and nuclear maturation [32]. If these physiological effects were applicable within an IVF population, it may be expected to lead to the increased recruitment of matured oocytes, and the improvement of oocyte developmental competence.

Supplementation with GH has been used as an IVF adjuvant therapy for decades, especially in patients with poor ovarian response (POR) [33–36]. Recently, several studies have found that adjuvant treatment of women with GH during controlled ovarian stimulation increases the number of oocytes collected, the embryo quality and the clinical pregnancy rate compared with untreated women [35–38]. These GH-mediated improvements may be related to the elevation of mitochondrial activity in the oocyte. Results from mechanistic studies have shown that GH can improve mitochondrial function of oocytes in patients with POR, in older women and in mice [39, 40], thereby increasing numbers of oocytes collected and embryo quality.

To date, only a few trials including observational and randomized controlled trials (RCTs), have been conducted to investigate the effects of GH on IVF treatment. A Hazout et al. demonstrated GH improved the number of oocytes collected and embryos obtained [37]. Meta-analysis found that GH increased the live birth rate in patients with POR [41, 42]. Recently, several studies found that GH promoted pregnancy rate or live birth rate by reducing miscarriage rates in POR patients [43, 44]. On the contrary, numerous other studies demonstrated that GH has no clear positive effect on pregnancy or live birth outcomes [45–47]. Most of the research on the effects of GH has focused on the treatment of POR, however, the effects of GH on patients with poor embryonic development remain unclear.

In this study, we performed an RCT to assess the effect of GH supplementation in IVF/ICSI patients with poor embryonic development. To determine whether this GH supplementation improved oocyte developmental competence, CGC mtDNA copy numbers were compared between patients with and without GH treatment.

## Methods

### Study period and participants

**Fig. 1:**
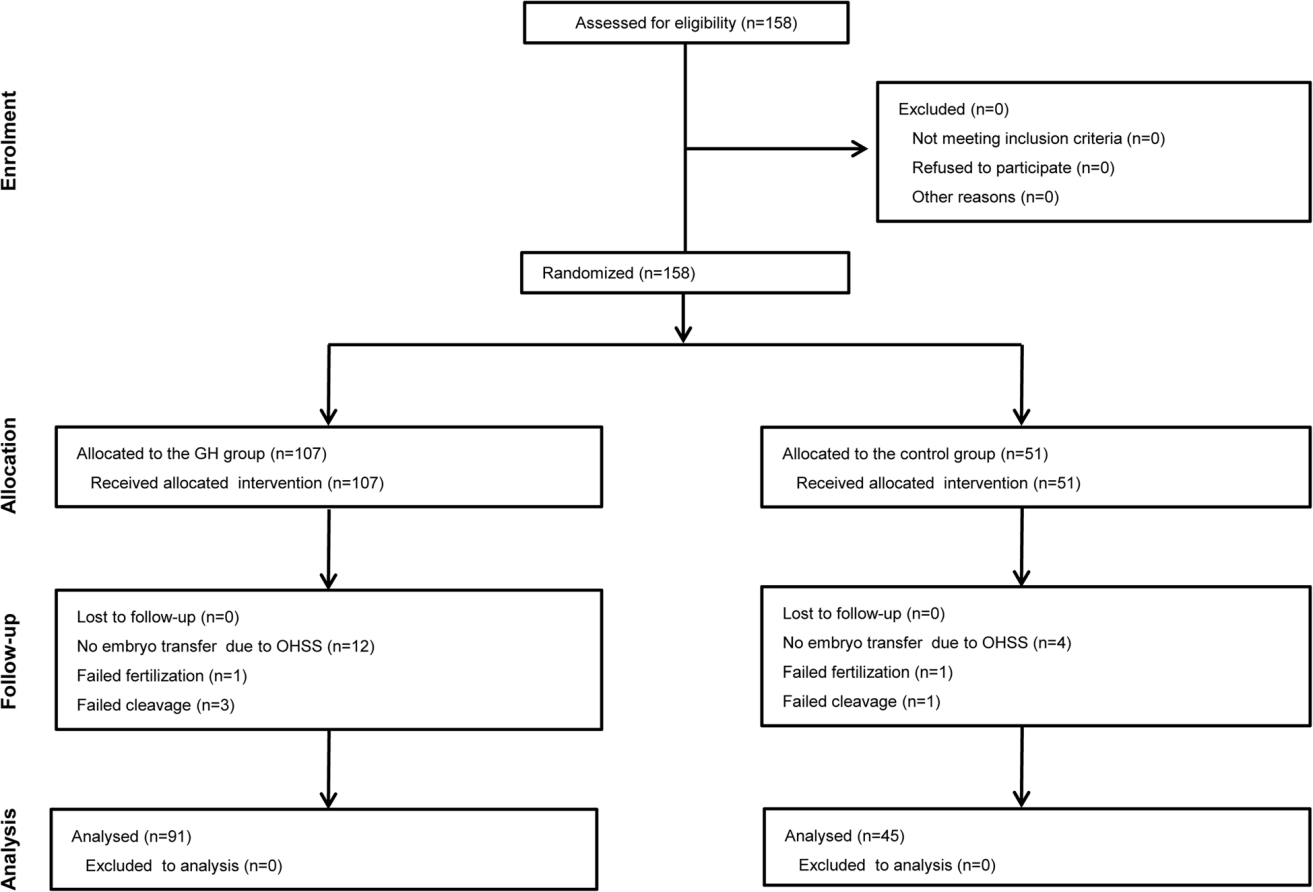
CONSORT statement flow diagram. GH, growth hormone; OHSS, ovarian hyperstimulation

This randomized, prospective study was registered retrospectively at ChiCTR (#1900021992), and conducted at the IVF centre of Chongqing Maternal and Child Health Care Hospital, Chongqing, China. We recruited patients in the study between July 2017 and February 2019 (Fig. 1). Randomization was carried out by computer, and female patients were assigned to the GH and control groups at a proportion of 2:1. The patients and doctors could not be blinded to the allocation because there was no placebo treatment for the control group, but the embryologists who graded the embryos were blinded. For clinical follow-up of the IVF outcome, serum concentrations of human chorionic gonadotropin (hCG) were measured 14 days after embryo transfer (ET). Clinical pregnancy was confirmed by the presence of a gestational sac in ultrasonographic examination at week 4. Pregnancy loss within 12 weeks was defined as early miscarriage. Pregnancy after early miscarriage was defined as ongoing pregnancy. Live birth rate was defined as the number of achieved live births after 28 weeks’ gestation.

Patients were recruited according to the following inclusion criteria: (1) at least one previous IVF cycle failure with no top-quality embryos (grade 1 or 2); (2) follicle-stimulating hormone (FSH), luteinizing hormone (LH), and oestradiol (E2) concentrations in the normal range during the early follicular phase; (3) aged 20–45-years old [48]; unexplained infertility with normal spermatozoa before IVF or subnormal spermatozoa justifying ICSI; (5) a normal uterine cavity with regular spontaneous menstrual cycles of 25–30 days; and [48] body mass index (BMI) < 25 kg/m^2^. The exclusion criteria were (1) any genetic disease; (2) polycystic ovarian syndrome (PCOS) or endometriosis; (3) azoospermia in the male partner; and [48] a history of endocrine disorders.

### Clinical management

Two ovarian-stimulation protocols were used: the gonadotropin-releasing hormone (GnRH) long agonist protocol and the GnRH antagonist protocols. The stimulation protocol was decided according to individual patient characteristics. Gonadotropins were prescribed at the discretion of the physicians in accordance with the results of ovarian-reserve tests (anti-Müllerian hormone, antral follicular count, basal FSH) and age. The dosage was then adjusted according to follicular growth until the day of administration of hCG.

The long protocol was performed as described previously [49]. Briefly, after downregulation with a GnRH agonist (triptorelin acetate, Ipsen Pharma, Paris, France), the ovaries were stimulated with gonadotropins. For the antagonist protocol, patients were stimulated with recombinant FSH using specific dosage algorithms.

Patients in the GH group received 3 IU recombinant human GH (Jintropin AQ, Gensci, Changchun, China) per day, from the initial day of downregulation for the long protocol or stimulation for the antagonist protocol until the day of the hCG trigger. The average duration of GH co-treatment was 25 days for patients receiving the long agonist protocol and 10 days for those receiving the antagonist protocol.

hCG (Ovidrel, Merck Serono, Switzerland) was administered when at least three follicles measured > 18 mm in diameter. Transvaginal oocyte retrieval was performed 36 h after hCG injection, and ET was performed on day 3 after oocyte retrieval.

### Isolation of oocytes and cumulus cells

Oocyte–cumulus complexes (OCCs) were retrieved 36 h after treatment with hCG and washed in multiple dishes with Flushing medium (OrigioFrance, Limonest, France) to eliminate the remaining mural granulosa cells, blood cells and cellular debris. The OCCs were subsequently cultured in G-IVF medium (Vitrolife, Kungsbacka, Sweden) for 2–3 h. For each OCCs, the CGCs were individually removed from OCCs via fine needles and gently pipetted with a 125-μm-diameter stripper pipette. CGCs were recovered in 500 μl of physiological serum and centrifuged at 10000 *g* for 5 min. The supernatant was removed, and the CGC pellets were immediately frozen at − 80 °C until they were processed for DNA extraction.

### DNA extraction

Total DNA extraction from isolated CGCs was carried out using the AllPrep DNA/RNA Micro Kit (Qiagen, Germantown, MD, USA) according to the manufacturer’s recommendations. Briefly, CGCs were first lysed and homogenized in a highly denaturing guanidine-isothiocyanate-containing buffer, which immediately inactivated DNases and RNases, to ensure the isolation of intact DNA and RNA. The lysate was then passed through an AllPrep DNA spin column. This column, in combination with the high-salt buffer, selectively and efficiently bound genomic DNA. The column was washed, and pure, ready-to-use DNA was eluted in 60 μl of water.

### Quantification of mtDNA

The mean mtDNA copy number in CGCs was determined by real-time quantitative PCR (Q-PCR) using SYBR green DNA intercalator and the CFX Connect system (Bio-Rad, Hercules, CA, USA). Primers were obtained from the Human Mitochondrial DNA (mtDNA) Monitoring Primer Set (Takara, Kusatsu, Japan). Briefly, two primer pairs (*ND1* and *ND5*) were used for the detection of mtDNA and two primer pairs (*SLCO2B1* and *SERPINA1*) for the detection of nuclear DNA. The mtDNA copy number was calculated twice, by determining the ratios for the *ND1*/*SLCO2B1* pair and *ND5*/*SERPINA1* pair. The final mtDNA copy number was determined as the average of these two calculated values.

### Embryo culture and assessment

For IVF procedures, oocytes were inseminated with motile spermatozoa at a concentration of 10,000 per ml. For ICSI, oocytes were microinjected with sperm 4–6 h after oocyte retrieval. The fertilized oocytes were transferred into pre-equilibrated culture dishes (Thermo Scientific, Waltham, MA, USA) with 25 μl of culture medium (Vitrolife Sweden, Gothenburg, Sweden) covered with 1.2 ml of paraffin oil (Vitrolife Sweden). The embryos were cultured in an incubator (MCO-5 M, Sanyo, Osaka, Japan) at 37 °C with 5% O_2_ and 6% CO_2_ until ET on day 3.

Embryo morphology was assessed according to the guidelines of the European Society of Human Reproduction and Embryology/Alpha consensus [50]. Embryos were graded according to blastomere number, blastomere size, and degree of fragmentation, using a morphological scoring system, accounting for the regularity of the blastomeres, degree of fragmentation, and microscopic appearance of the embryos. Transferable embryos were defined by the presence of 6–10 symmetrical blastomeres and < 20% fragmentation, with no multinucleation, on day 3.

### Sample-size determination and statistical analysis

The primary outcome was the clinical pregnancy rate, and in the pre-trial period, clinical pregnancy rates were approximately 20 and 41% in untreated and GH-treated women, respectively. The difference between these rates was used for sample-size calculation, which, with 70% power and a group-size ratio of 1:2, indicated that 99 and 50 patients were needed in the GH and control groups, respectively.

Continuous variables are presented as the mean ± standard deviation. Categorical variables are presented as *n* (%). For comparisons between the groups, χ^2^ test exact test was used for dichotomous variables, and the Student’s *t* test was used for continuous variables. For main variables, 95% confidence intervals (CIs) were determined for the mean difference (MD) and relative risk (RR) in the estimates. A *P*-value < 0.05 was considered significant. All statistical tests were performed using SAS software version 9.3 (SAS Institute, Cary, NC, USA) and SPSS software version 22, 2013 (SPSS, Chicago, IL, USA).

## Results

### Overview of patient characteristics and ovarian response

The flow chart for patient recruitment in this study is shown in Fig. 1. We recruited 158 patients and randomized them into GH (*n* = 107) and control (*n* = 51) groups after recruitment between July 2017 and February 2019. None of the patients was lost to follow-up. In the two groups, two cases and four cases were canceled because of fertilization and cleavage failure, due to low numbers of retrieved oocytes. There were 16 cases without embryo transfer in the two groups, all due to ovarian hyperstimulation.

Patient characteristics are presented in Table 1. No significant differences were found between the two groups in terms of age, infertility duration, BMI, AMH, day 3 serum levels of FSH, LH, E2, and progesterone (P), duration of stimulation, total gonadotropin dose, or endometrial thickness.

**Table 1:** Comparisons of baseline characteristics of female participants in the growth-hormone (GH)-treated and control groups

	GH (*n* = 107)	Control (*n* = 51)
Age (years)	32.96 ± 4.67	32.86 ± 4.32
Infertility (years)	6.21 ± 3.77	6.35 ± 4.64
Body mass index (kg/m^2^)	22.09 ± 2.71	21.93 ± 2.66
Anti-Mullerian hormone (ng/ml)	2.61 ± 2.05	2.38 ± 2.03
Follicle-stimulating hormone (mIU/ml)	5.79 ± 2.46	6.09 ± 2.33
Luteinizing hormone (mIU/ml)	2.99 ± 1.36	3.13 ± 1.88
Oestradiol (pg/ml)	33.69 ± 16.20	33.55 ± 20.08
Progesterone (mIU/ml)	0.31 ± 0.16	0.31 ± 0.14
Days of gonadotropins (days)	10.11 ± 1.42	10.45 ± 1.94
Total gonadotropin dose (IU)	2402.14 ± 648.33	2604.41 ± 853.42
Endometrial thickness (mm)	9.62 ± 1.88	9.97 ± 1.92
Stimulation protocols		
Antagonist	27/107 (25.23%)	11/51 (21.57%)
Long protocol	80/107 (74.77%)	40/51 (78.43%)

All variables are presented as mean ± SD. For comparisons between the groups, the Student’s *t* test was used

### Comparison of embryological and clinical data between the GH and control groups

Relative to the control group, the GH group had a significantly higher number of retrieved oocytes, number of metaphase II (MII) oocytes, number of 2PN fertilized oocytes, and number of cleaved embryos on day 2 (Table 2). Although the GH group also had a slightly higher number of transferable embryos than the control group, this difference was not statistically significant (Table 2).

**Table 2:** Comparison of embryology data between the growth-hormone (GH)-treated and control groups

	GH (*n* = 107)	Control (*n* = 51)	*P*-value	MD (95% CI)
No. of oocytes retrieved per patient	10.29 ± 5.92	8.16 ± 4.17	0.022^a^	2.130 (0.528–3.732)
No. of metaphase II oocytes	9.08 ± 5.83	7.49 ± 3.98	0.040^a^	1.590 (0.036–3.144)
No. of 2PN oocytes	6.77 ± 4.51	5.45 ± 3.30	0.049^a^	1.320 (0.075–2.565)
Fertilization rate (%)	724/972 (74.49%)	278/382 (74.73%)	0.564^b^	
No. of cleaved embryos	6.73 ± 4.25	5.29 ± 3.23	0.033^a^	1.440 (0.242–2.638)
Cleavage rate (%)	693/724 (95.72%)	270/278 (97.12%)	0.397^b^	
No. of transferable embryos	3.30 ± 2.14	2.96 ± 1.97	0.400^a^	
Transferable embryo rate (%)	340/693 (49.06%)	151/270 (55.93%)	0.065 ^b^	
No. of transferred embryos per ET	1.92 ± 0.40	1.87 ± 0.40	0.464^a^	
Cycles reaching ET rate (%)	91/107 (85.05%)	45/51 (88.24%)	0.768 ^b^	
Reason for lack of ET				
OHSS, freezing of all embryos, *n*	12	4		
Failed fertilization, *n*	1	1		
Failed cleavage, *n*	3	1		

Categorical variables are presented as proportion (%). Continuous variables are presented as mean ± SD

For comparisons of dichotomous variables, χ^2^ test was used. For comparisons of continuous variables, Student’s *t* test was used

*CI* confidence interval, *ET* embryo transfer, *MD* mean difference, *OHSS* ovarian hyperstimulation

Fertilization rate: No. of 2PN oocytes/ No. of metaphase II oocytes; Cleavage rate: No. of cleaved embryos/ No. of 2PN oocytes; Transferable embryo rate: No. of transferable embryos/ No. of cleaved embryos

^a^, Student’s *t* test; ^b^, χ^2^ test

There was no significant difference between GH and control group with regard to the fertilization rate, cleavage rate, transferable embryo rate, cycles reaching ET rate and number of transferred embryos per ET (Table 2). However, The GH group had significantly improved clinical outcomes relative to the control group, including implantation rate, clinical pregnancy rate, ongoing pregnancy rate, live birth rate per cycle start or per ET (Table 3). The relative risk (95% confidence interval) was 2.324 (1.299–4.158) for the clinical pregnancy rate per ET and 2.418 (1.298–4.502) for the live birth rate per ET, indicating a significant effect for using GH. Furthermore, the significantly higher ongoing pregnancy rate and live birth rate in both fresh and frozen cycles in GH group were also detected. No adverse events were associated with the use of GH in this study.

**Table 3:** Comparison of clinical data between the growth-hormone (GH)-treated and control groups

	GH (*n* = 107)	Control (*n* = 51)	*P*-value	RR (95% CI)
Implantation rate (%)	63/175 (36.00%)	15/84 (17.86%)	0.005	2.016 (1.224–3.322)
Clinical pregnancy rate/cycle start (%)	47/107 (43.93%)	10/51 (19.61%)	0.005	2.240 (1.235–4.064)
Clinical pregnancy rate/ET (%)	47/91 (51.65%)	10/45 (22.22%)	0.002	2.324 (1.299–4.158)
Early miscarriage rate/cycle start (%)	3/107 (2.80%)	1/51 (2.00%)	1	
Early miscarriage rate//ET (%)	3/91 (3.30%)	1/45 (2.22%)	1	
Fresh ET rate (%)	69/91 (75.82%)	37/45 (82.22%)	0.531	
Fresh ET clinical pregnancy rate (%)	37/69 (53.62%)	8/37 (21.62%)	0.003	2.480 (1.293–4.758)
Fresh ET ongoing pregnancy rate (%)	34/69 (49.28%)	7/37 (18.92%)	0.004	
Fresh ET live birth rate (%)	34/69 (49.28%)	7/37 (18.92%)	0.004	2.605 (1.282–5.291)
Frozen ET rate(%)	22/91 (24.18%)	8/45 (17.78%)	0.531	
Frozen ET clinical pregnancy rate (%)	10/22 (45.45%)	2/8 (25.00%)	0.555	
Frozen ET ongoing pregnancy rate (%)	10/22 (45.45%)	2/8 (25.00%)	0.555	
Frozen ET live birth rate (%)	10/22 (45.45%)	2/8 (25.00%)	0.555	1.818 (0.503–6.569)
Ongoing pregnancy rate /cycle start (%)	44/107 (41.12%)	9/51 (17.65%)	0.006	
Ongoing pregnancy rate /ET (%)	44/91 (48.35%)	9/45 (20.00%)	0.003	
Live birth rate/cycle start (%)	44/107 (41.12%)	9/51 (17.65%)	0.006	2.330 (1.235–4.396)
Live birth rate/ET (%)	44/91 (48.35%)	9/45 (20.00%)	0.003	2.418 (1.298–4.502)

Categorical variables are presented as proportion (%)

For comparisons of dichotomous variables, χ^2^ test was used

*CI* confidence interval, *ET* embryo transfer, *RR* relative risk

### Comparisons of CGC mtDNA copy number

**Fig. 2:**
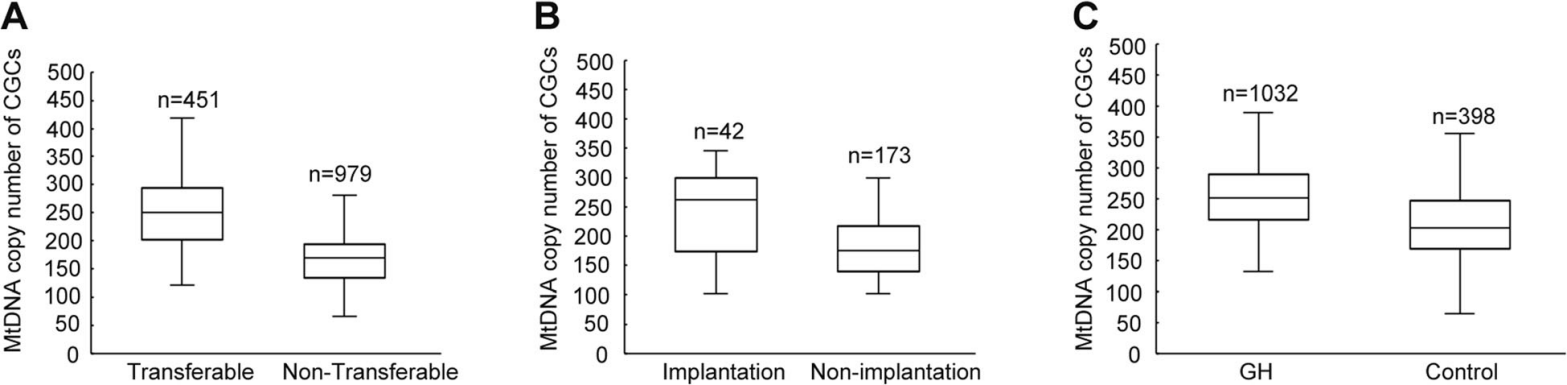
The relationships between CGC mtDNA and embryo quality, implantation, and GH supplementation. **a** mtDNA copy number per CGC for transferable and non-transferable embryos. **b** mtDNA copy number per CGC for implanted and non-implanted embryos. **c** mtDNA copy number per CGC for the GH and control groups. CGC, cumulus granulosa cell; GH, growth hormone; mtDNA, mitochondrial DNA

mtDNA was quantified from a total of 1430 CGCs that were obtained from the 158 female participants. The median mtDNA copy number in the CGCs of transferable embryos derived from the control group and the two groups were both significantly higher than that of non-transferable embryos (250 versus 169, *P* < 10^− 4^) (Fig. 2a and Figure S1). Furthermore, the mtDNA copy number in CGCs was significantly higher for the simultaneous implantation of two embryos than for the non-implanted group (262 versus 175, *P* < 10^− 4^) (Fig. 2b). Then we investigated the effect of GH supplementation on oocyte quality in the two patient groups (using mtDNA copy number as a proxy), we found a significantly higher copy number in CGCs in the GH group (252 versus 204, *P* < 10^− 4^) (Fig. 2c), suggesting that GH treatment in IVF/ICSI cycles increase the mitochondrial activity in oocytes.

## Discussion

GH supplementation is used as an adjuvant therapy for patients with POR or older age who undergo IVF [51]. In this study, we examined the effect of adding GH to the GnRH antagonist protocol or the GnRH agonist long protocol in 158 women with poor embryonic development who underwent IVF/ICSI cycles. We found that the numbers of oocytes retrieved, 2PN fertilized oocytes, and cleaved embryos on day 2 were significantly higher in the GH group than in the control group. Most importantly, we also identified increased clinical pregnancy and live birth rates in the GH group.

To the best of our knowledge, this study is the first in which the impact of GH co-treatment during IVF/ICSI cycles in women with poor embryonic development has been investigated. In our study cohort, there was no significant difference in the total gonadotropin dose or mean E2 level on the trigger day between the two groups, in contrast to previous findings [33, 38]. A possible explanation for this discrepancy is that high GH doses (8 IU/day and 12 IU/day) were used in the previous two studies, whereas we used a low dose (3 IU/day) in the short-term stimulation cycle, because increasing the dose would have added substantially to the cost of the trial. We did not find a significant difference in the total number of days of gonadotropin stimulation between the two groups, whereas in a European and Australian multi-centre study of women with hypogonadotropic hypogonadism [52], GH treatment resulted in a significant dose-dependent decrease in ovarian stimulation time.

Several studies have demonstrated that adjuvant GH treatment can improve the IVF/ICSI outcome in patients with POR, via elevation of the number of oocytes (including MII oocytes) that are retrieved [33, 37, 38, 53], and in turn the fertilization rates and the numbers of transferable embryos. Our results corroborated these previous findings, demonstrating that adjuvant GH treatment in women with history of poor embryonic development increased the number of oocytes (including 2PN fertilized oocytes) that were retrieved, compared with the control group. However, although we observed a higher number of transferable embryos in the GH group than in the control group, this difference was not statistically significant. Although it is possible that, with a larger sample size, this effect might become significant, the observed difference in the number of transferable embryos was not sufficient to account for the greater than twofold increase that occurred in implantation rate, clinical pregnancy rate, and live birth rate. Thus, we speculate that GH supplementation improves the true developmental competence of oocytes, rather than simply increasing the number of retrieved oocytes.

Results from human and animal studies have demonstrated that GH has important roles in the processes of folliculogenesis, steroidogenesis, and oocyte maturation [54]. GH can stimulate the production of insulin-like growth factor 1 (IGF-1) in serum and follicles [28]. In animal models, IGF-1 can suppress follicular apoptosis [55]. Avoidance of apoptosis is also essential for follicular development and oocyte maturation [56]. Overall, this suggests that GH acts through IGF-1 to promote follicular development and the inhibition of follicular apoptosis. The mechanism underlying GH-mediated improvement of oocyte quality is presumed to be the elevation of oocyte mitochondrial function [39, 40, 57]. The mitochondrial activity in oocytes decreases with age [58]. During oocyte maturation, interactions with CGCs ensure that the oocytes have sufficient energy-production reserves for meiosis and to support subsequent embryonic development. Here, we confirmed that the mtDNA content in CGCs is positively associated with corresponding embryo development and implantation competence, which is consistent with previous observations [23, 24]. Therefore, we can use the mtDNA content in CGCs as a reliable biomarker for the assessment of oocyte quality.

Several studies have demonstrated that GH can improve the mitochondrial activity of oocytes. However, majority of these studies focused on the effect of GH on the older patients, and the results were from in vitro matured oocytes or animal models [39, 40, 57]. Until now, there is no research including large data of mtDNA copy number of CGCs in normal human oocytes for the assessement of the effect of GH on the oocyte quality, especially in patients with poor embryonic development. In the present study, we calculated the mtDNA copy number in total 1430 CGCs in GH and control group. The results showed that the mtDNA copy number of CGCs was significantly higher in the GH group than in the control group, suggesting that adjuvant GH treatment in IVF/ICSI cycles does indeed improve oocyte developmental competence.

The quality of the transferred embryo is a key factor in successful implantation and pregnancy, as confirmed by our results. However, we did not detect a change in the number of transferable embryos in response to GH treatment, possibly because improvements in features that affect the quality of the embryo, such as metabolism, ATP homeostasis, and gene expression, cannot be detected through morphological assessment. Rather, the higher quality of the transferred embryos in the GH group was demonstrated by the more-than-twofold improvement in the clinical pregnancy and live birth rates compared with the control group, indicating the potential benefits of GH treatment in patients with poor embryonic development.

Our study had some notable limitations. We classified patients with previous implantation failure that was mainly defined by the absence of top-grade embryos (grade 1 or 2) as patients with ‘poor embryonic development’ (noting that there is no standard definition for this group of patients). However, poor embryonic development in 1 cycle does not necessarily mean it will also occur in the next cycle. To address this issue, we plan to recruite only patients with three or more previous failures resulting from absence of top-grade embryos in later study, which require more than 5 years for the recruitment of a sufficient number of such participants, because these patients represent a small fraction of patients undergoing IVF. Another limitation of the study is that we were not able to assess the cumulative live birth rate, because not all patients had all of their cryopreserved embryos transferred. However, we also can demonstrate the benefits of GH treatment in IVF/ICSI cycles from the improvements in the live birth rate and mtDNA copy number of CGCs.

## Conclusions

The results of this study have demonstrated that GH supplementation may have a positive effect on IVF outcome, including improvements in the numbers of oocytes retrieved and live birth rate, in patients with poor embryonic development. Furthermore, the increased CGC mtDNA copy number in GH group provide an indirect evidence that GH con-treatment may improve the oocyte quality and developmental competence.

## Supplementary information

**Additional file 1: Figure S1.** The relationships between CGC mtDNA and embryo quality and implantation in control group. (**A**) mtDNA copy number per CGC for transferable and non-transferable embryos. (**B**) mtDNA copy number per CGC for implanted and non-implanted embryos.

## Data Availability

The datasets used and analyzed in the current study are available from the corresponding author on reasonable request.

## Abbreviations

GH: Growth hormone
IVF: In vitro fertilization
mtDNA: Mitochondrial DNA
CGCs: Cumulus granulosa cells
OET: Oocyte-to-embryo transition
ATP: Adenosine triphosphate
POR: Poor ovarian response
RCT: Randomized controlled trial
hCG: Human chorionic gonadotropin
ET: Embryo transfer
FSH: Follicle-stimulating hormone
LH: Luteinizing hormone
E2: Oestradiol
BMI: Body mass index
PCOS: Polycystic ovarian syndrome
GnRH: Gonadotropin-releasing hormone
OCCs: Oocyte–cumulus complexes
CIs: Confidence intervals
MD: Mean difference
RR: Relative risk
OHSS: Ovarian hyperstimulation
