# Supplementary figures and images for: Does growth hormone supplementation improve oocyte competence and IVF outcomes in patients with poor embryonic development? A randomized controlled trial
Source: BMC Pregnancy Childbirth. 2020 May 20;20:310. doi: 10.1186/s12884-020-03004-9 (PMC7238549; doi:10.1186/s12884-020-03004-9)

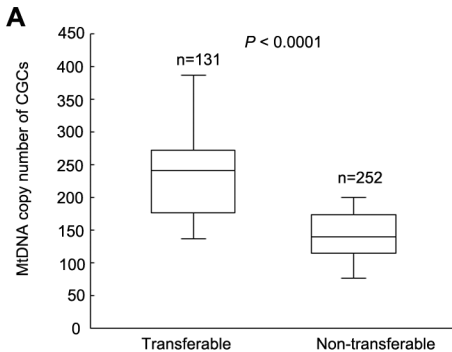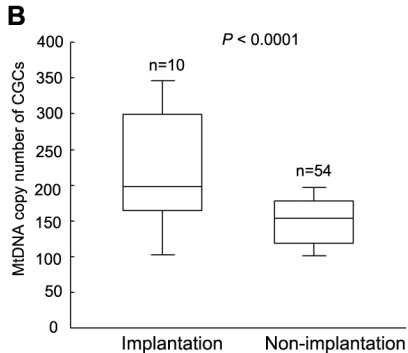

Supplement: Supplementary file 1 — Additional file 1: Figure S1. The relationships between CGC mtDNA and embryo quality and implantation in control group. (A) mtDNA copy number per CGC for transferable and non-transferable embryos. (B) mtDNA copy number per CGC for implanted and non-implanted embryos. [file 12884_2020_3004_MOESM1_ESM.pdf]
